# Noise within: Signal-to-noise enhancement via coherent wave amplification in the mammalian cochlea

**DOI:** 10.1103/physrevresearch.6.013084

**Published:** 2024-01-23

**Authors:** Alessandro Altoè, Christopher A. Shera

**Affiliations:** Auditory Research Center, Caruso Department of Otolaryngology, University of Southern California Los Angeles, Los Angeles, California 90033, USA

## Abstract

The extraordinary sensitivity of the mammalian inner ear has captivated scientists for decades, largely due to the crucial role played by the outer hair cells (OHCs) and their unique electromotile properties. Typically arranged in three rows along the sensory epithelium, the OHCs work in concert via mechanisms collectively referred to as the “cochlear amplifier” to boost the cochlear response to faint sounds. While simplistic views attribute this enhancement solely to the OHC-based increase in cochlear gain, the inevitable presence of internal noise requires a more rigorous analysis. Achieving a genuine boost in sensitivity through amplification requires that signals be amplified more than internal noise, and this requirement presents the cochlea with an intriguing challenge. Here we analyze the effects of spatially distributed cochlear-like amplification on both signals and internal noise. By combining a straightforward mathematical analysis with a simplified model of cochlear mechanics designed to capture the essential physics, we generalize previous results about the impact of spatially coherent amplification on signal degradation in active gain media. We identify and describe the strategy employed by the cochlea to amplify signals more than internal noise and thereby enhance the sensitivity of hearing. For narrow-band signals, this effective, wave-based strategy consists of spatially amplifying the signal within a localized cochlear region, followed by rapid attenuation. Location-dependent wave amplification and attenuation meet the necessary conditions for amplifying near-characteristic frequency (CF) signals more than internal noise components of the same frequency. Our analysis reveals that the sharp wave cutoff past the CF location greatly reduces noise contamination. The distinctive asymmetric shape of the “cochlear filters” thus underlies a crucial but previously unrecognized mechanism of cochlear noise reduction.

## I. INTRODUCTION

In the 19th century, Bernhard Riemann made the remarkable observation that the sound of a foghorn could be heard from a distance of five miles. He concluded that the human ear must be capable of detecting sounds that generate only subatomic motions of the eardrum [1]. During the succeeding one and a half centuries, Riemann’s conjecture has been repeatedly verified [2]. The extraordinary sensitivity of the mammalian ear can be attributed to the coordinated, piezoelectric behavior of outer hair cells (OHCs) [3]. Arranged in rows along the sensory tissue (the organ of Corti), these cells act as actuators capable of boosting sound-induced vibrations of the sensory tissue by more than two orders of magnitude [4]. The prevailing belief in the field posits that OHCs actively amplify sound-induced waves as they propagate along the spiral structure of the cochlea. Collectively, the mechanisms involved are known as the “cochlear amplifier.”

However, whether cochlear amplification constitutes a viable strategy for enhancing the sensitivity of hearing remains controversial. Because the minimum signal level to which sensory neurons can meaningfully respond is inherently limited by the level of internal noise (see, e.g., Ref. [5]), it remains unclear how the cochlear amplifier, while amplifying signals, can avoid amplifying the accompanying internal noise [6]. Although the dominant sources of intracochlear mechanical noise remain to be firmly identified—these necessarily include both thermal noise and mechanical noise generated by stochastic gating of hair-cells ion channels (see, e.g., Refs. [5–7])—intracochlear mechanical noise is both present and measurable, and it depends on the same mechanisms that control signal amplification [8]. While previous work has focused on the effects of noise on the mechanical sensitivity of inner-hair-cell stereocilia (see, e.g., Refs. [5,7]), propagation and amplification of intracochlear mechanical noise remains unexplored.

In this study, we investigate the impact of spatially distributed amplification on both signals and internal noise using two distinct but complementary approaches: a mathematical model of spatially distributed amplification and an active model of the cochlea. We begin by examining the simplest scenario, which involves a highly anisotropic, one-dimensional (1D) medium comprising a series of cascaded “noisy” amplifiers in which signals and noise propagate in only a single direction. We then move to the more challenging but biologically relevant case where the medium is nearly isotropic, so that signals and noise propagate and are amplified in both directions. Finally, we investigate signal and noise amplification within a simplified but physically realistic linear model of the cochlea. Importantly, our analysis concerns only noise sources that are located within the cochlea: The ear processes external noise in the same way that it processes signals [9]. Furthermore, as we all know from cocktail parties, which sounds are “signals” and which are “noise” depends entirely on what one wants to listen to.

## II. SPATIALLY DISTRIBUTED AMPLIFICATION IN NOISY ACTIVE MEDIA

### A. Propagation of signals and noise in one direction

**FIG. 1.**
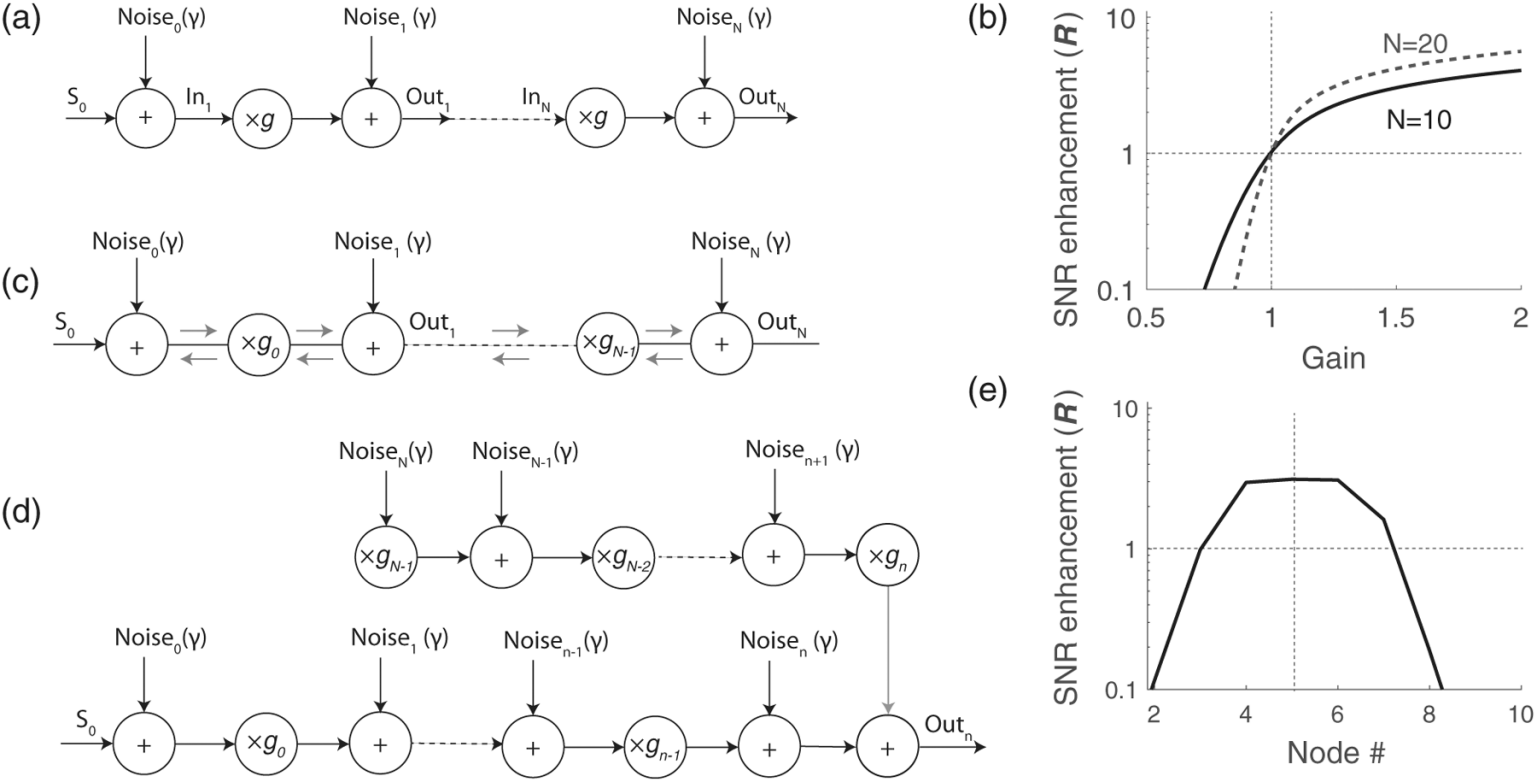
(a) Effect of spatially distributed “one-way” amplification on signal and internal noise. The model consists of a chain of linear amplifiers (multipliers) with gain g; the effect of internal noise is simulated by adding noise before and after each amplification stage. (b) SNR enhancement (**R**) at the Nth node of the amplifier chain (shown for N=10 and N=20) as a function of the amplifier gain, g. (c) Bidirectional noisy amplification model. In this model, internal noise propagates and is amplified identically in both directions. (d) Equivalent one-way amplification model to the study noise and signal response at the nth node. (e) Example of enhancement factor at different nodes in a chain of N=10 bidirectional amplifiers. In this example, the amplifier gain is chosen to improve the SNR at node 5 (see text) by setting $g_{m}=3$ for m<5 and $g_{m}=0.1$ for m⩾5.

We start by considering the simple scenario of the distributed “one-way” noisy amplifier, depicted in Fig. 1(a). The model consists of a chain of amplifiers that multiply the input signal (S[0]) by a factor g, representing the amplifier gain. The medium’s noise is represented by noise sources that are summed with the propagating signal after each amplification stage. To remove the ambiguity regarding whether noise should be included before or after the amplification stage, the model includes noise sources located both at the input of the first amplifier and at the output of last. This model approximates a strongly anisotropic medium, where signals and noise propagate only in one direction [from left to right in Fig. 1(a)]. This scenario accurately represents what occurs in many human-made systems, such as cascaded electronic amplifiers or radio repeaters—indeed, the formulae we derive here are essentially the same used to calculate the noise figure of cascaded electronic amplifiers [10].

In this model we can turn amplification “off”—and thereby model signal propagation in a lossless, noisy medium—by imposing the condition g=1. Or we can turn it “on” by setting g≠1. When g>1, the chain amplifies signals as they propagate. When g<1, the distributed amplifiers become distributed, attenuating “brakes.” By comparing signal and noise for the three conditions (g=1,g>1, and g<1), we quantify the impact of amplification and attenuation on the signal-to-noise ratio (SNR) along the chain [i.e., at the nodes Out_1,2…n_ in Fig. 1(a)].

The root-mean-square (rms) amplitude of the signal at a given node n is simply the rms amplitude of the input signal passed through n multipliers $\left(S_{rms}[n]=g^{n}S_{rms}[0]\right.)$. Turning on the amplifier thus boosts the signal amplitude by the factor

(1) $$G_{\text{signal }}\left[n\right]=g^{n}.$$

We focus our analysis on the physically relevant case where the noise sources are uncorrelated, meaning that the noise in the medium is spatially incoherent. For simplicity, we assume that the various noise sources are independent versions of the same stochastic process, with rms amplitude γ. In this case, the rms amplitude of the noise $\left(N_{\text{rms}}\right)$ at node n can be calculated by incoherent summation (i.e., linear summation of power) of the various amplified noise terms. Specifically, the noise power at node n can be expressed as a geometric series, where the m-th term represents the contribution of the (n-m)-th source, amplified (or attenuated) m times. The expression for $N_{rms}[n]$ can be simplified based on different scenarios:

(2) $$N_{\text{rms}}\left[n\right]=\sqrt{\overset{n}{\underset{m=0}{\sum }}g^{2m}\gamma }=\{\begin{array}{ll} \sqrt{\frac{g^{2\left(n+1\right)}-1}{g^{2}-1}}\gamma & \text{ for }g\neq 1,\\ \sqrt{n+1}\gamma & \text{ for }g=1. \end{array}$$

Hence, turning on the amplifier boosts the noise gain by a factor of

(3) $$G_{\text{noise }}\left[n\right]=\frac{{N_{\text{rms}}\left[n\right]|}_{g\neq 1}}{{N_{\text{rms}}\left[n\right]|}_{g=1}}=\sqrt{\frac{g^{2\left(n+1\right)}-1}{\left(n+1\right)\left(g^{2}-1\right)}}.$$

The SNR at node n is given by $R_{n}=S_{\text{rms}}[n]/N_{\text{rms}}[n]$. The effect of amplification on the system’s sensitivity can be quantified by the SNR enhancement factor [11]:

(4) $$R\left[n\right]=R_{n}\left(\text{on }\right)/R_{n}\left(\text{off }\right)=G_{\text{signal }}/G_{\text{noise }},$$

where R(on) and R(off) are the SNR with the amplifier on (g≠1) and off (g=1), respectively. Figure 1(b) illustrates the enhancement factor as a function of g for two values of n. When R>1 the signal is amplified more than the internal noise, and the SNR increases at the considered node. Conversely, when R<1, the signal is amplified less than the noise, and the SNR decreases. It follows from Eqs. (1) and (3) that amplification (g>1) boosts signals more than internal noise, increasing the SNR at all nodes. In particular, the larger the gain, the larger R, resulting in a greater improvement in SNR at any node. Additionally, the longer the chain of amplifiers, the larger the benefit of distributed amplification on the SNR and the greater the increase in the system’s sensitivity. Conversely, when the amplifiers act as attenuators (g<1), R<1, meaning that the signal is attenuated more than the internal noise.

As the signal propagates along the line, noise from the growing number of contributing sources accumulates. A relevant measure of the resulting signal degradation is the noise factor $F_{n}=R_{n}/R_{0}$, which quantifies how the SNR degrades along the transmission line. In our case

(5) $$F_{n}=\sqrt{\frac{g^{2\left(n+1\right)}\left(1-g^{-2}\right)}{g^{2\left(n+1\right)}-1}},$$

which approaches 1 (i.e., no significant SNR degradation along the line) when g≫1. Importantly, this result—namely that distributed amplification prevents signal degradation—generalizes to the case when internal noise sources are spatially coherent [12].

### B. Signal vs noise amplification in isotropic active media

We now extend the simple chain-of-amplifiers model described above by considering the case of an active medium where waves propagate in both directions, as in the mammalian cochlea [13]. In our simplified treatment, we assume that the medium is isotropic. Thus, we assume that the amplifiers boost signals propagating in either direction by the same amount [Fig. 1(c)]. We simplify the analysis further by ignoring potential scattering effects within the medium and by assuming that the various noise sources all have equal amplitudes. In this case, however, we allow the amplifier gain to vary along the line. When considering signal and noise propagation to node n, the system can be depicted as the combination of two “one-way” amplification models [Fig. 1(d)], representing the contribution from sources located to the right and to the left of the node n. Note that whereas signals come only from the left, noise comes from both directions.

Signal propagation from a source node $n^{\prime }$ to a receiver node n is encapsulated by the discrete Green’s function $G\left[n,n^{\prime }\right]$. In the simplified model, where each node n amplifies the signal by the factor $g_{n}$:

(6) $$G\left[n,n^{\prime }\right]=\overset{\text{max}\left(n,n^{\prime }\right)-1}{\underset{m=\text{min}\left(n,n^{\prime }\right)}{\prod }}g_{m}.$$

Note that the Green’s function is symmetric: $G\left[n^{\prime },n\right]=G\left[n,n^{\prime }\right]$. In this model, the signal is effectively a source at node 0; its amplitude at node n is therefore

(7) $$S_{\text{rms}}\left[n\right]=S_{\text{rms}}\left[0\right]G\left[n,0\right].$$

The noise response at node n can be decomposed into the incoherent summation of noise from both the left and right sides of the node [Fig. 1(d)]:

(8) $$N_{\text{rms}}\left[n\right]=\sqrt{\overset{N}{\underset{n^{\prime }=0}{\sum }}{\left(G\left[n,n^{\prime }\right]\gamma \right)}^{2}}\;=\gamma \sqrt{\overset{n}{\underset{n^{\prime }=0}{\sum }}{\left(G\left[n,n^{\prime }\right]\right)}^{2}+\overset{N}{\underset{n^{\prime }=n+1}{\sum }}{\left(G\left[n,n^{\prime }\right]\right)}^{2}}.$$

In this case, unlike the simpler anisotropic model of Fig. 1(a), amplification is not necessarily beneficial for the SNR. When the goal is to maximize the SNR at node n, the optimal gain distribution along the amplifier chain is

(9) $$g_{n^{\prime }}\gg 1\text{ for }n^{\prime }<n\;g_{n^{\prime }}\ll 1\text{ for }n^{\prime }⩾n.$$

In this case, the system approaches the performance of the one-way amplification model at the nth node. Unlike the one-way model, however, it is not possible to increase the SNR at all nodes simultaneously [see Fig. 1(e)].

## III. SIGNAL-VS-NOISE AMPLIFICATION IN THE MAMMALIAN COCHLEA

### A. Preliminaries

**FIG. 2.**
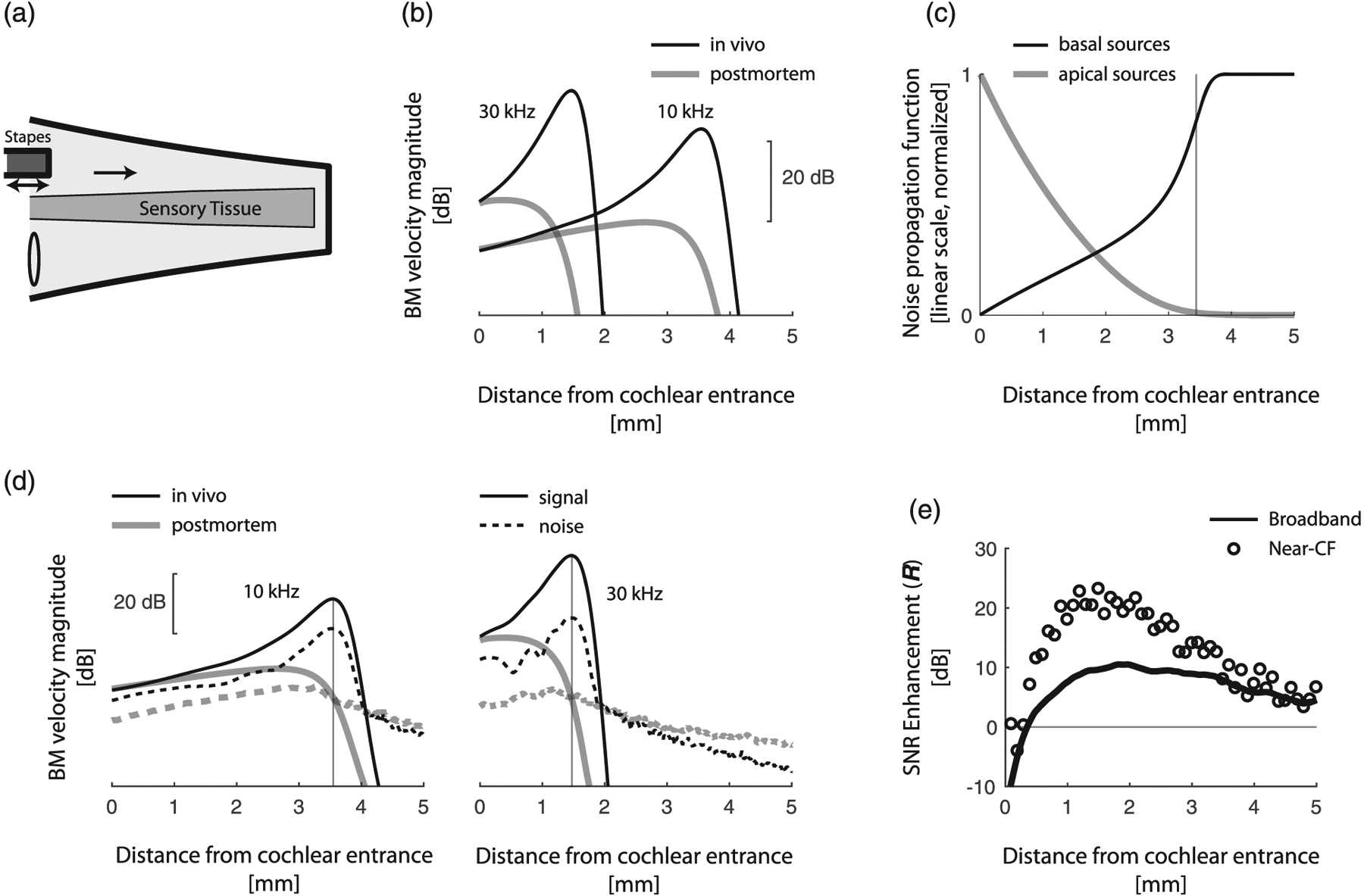
(a) Simplified anatomical view of the mammalian cochlea. (b) BM magnitude responses *in vivo* (amplifier on) and postmortem (amplifier off) to stimulus tones of 10 kHz and 30 kHz calculated in a 2D finite-difference model of the mouse cochlea. (c) Apical and basal noise propagation functions for narrow-band noise centered around 10 kHz. At each location, these functions quantify the expected noise power due to distributed basal and apical noise sources of equal strength, respectively. The gray vertical line marks the characteristic place. (d) BM response magnitude to sound signal and narrow-band internal noise at 10 and 30 kHz for both postmortem and *invivo* models. The curves are normalized so that the signal and noise magnitudes at the characteristic places (vertical gray lines) are the same postmortem. The difference between *in vivo* signal and noise responses demonstrates that turning on the amplifier boosts the SNR at the characteristic place. (e) Enhancement factor (i.e., the ratio between the SNR with the amplifier on and the amplifier off) along the cochlea calculated for narrow-band near-CF signals and noise and for broadband signals and noise (assumed white over the band from 4 to 70 kHz). The figure shows that the near-CF positive SNR enhancement caused by turning on the amplifier produces a global, broadband increase in SNR.

Figures 2(a) and 2(b) illustrate the general function of the mammalian ear. Briefly, sound-induced vibration of the stapes (the third of the three middle-ear ossicles in the chain that connects the eardrum to the cochlea) displaces the fluid in the inner ear, launching hydromechanical waves that propagate slowly from the base (i.e., the entrance) toward the apex (i.e., the “end”) of the cochlea. Cochlear wave propagation is frequency dependent, so that waves peak on the BM at locations that depend on frequency. In this way, the cochlea maps frequency into position, with higher frequencies mapping closer to the stapes. As they travel apically beyond their peak location, cochlear waves are dramatically attenuated. Cochlear wave propagation is also nonlinear (intensity dependent) and varies with cochlear health [e.g., *in vivo* vs postmortem, see Fig. 2(b)]. In particular, the location of maximal vibration depends both on sound level and on physiological status. However, at sound levels near the threshold of hearing, where issues concerning SNR are most pressing, cochlear mechanical responses are approximately linear. For this reason, we employ linear models for our analysis. At any location we define the characteristic frequency (CF) as the frequency that evokes the largest *in vivo* BM response at low sounds levels; conversely, we define the characteristic place as the location where a wave of given frequency peaks on the BM at low sound levels.

*In vivo*, the cochlear amplifier boosts waves as they propagate towards their characteristic places, producing stronger and more spatially localized responses than in a dead cochlea [Fig. 2(b)]. Equivalently, because of the well-established symmetry between spatial and frequency tuning [14], the cochlear amplifier narrows the bandwidth of BM frequency responses measured at a given location (colloquially, these frequency responses are known as “cochlear filters”). By narrowing the bandwidth of the cochlear filters, amplification enhances cochlear sensitivity through well-known principles [15]. Indeed, narrowing the bandwidth of a receiver means reducing its response to background broadband noise relative to the response to a signal within the receiver passband. However, because it is theoretically possible to narrow the bandwidth of the cochlear filters without resorting to amplification (e.g., Ref. [16]), we make a dedicated effort to isolate the effects of signal amplification from the effects of amplifier-induced bandwidth reduction.

### B. Cochlear amplification

In our analysis of cochlear mechanics, we consider a general linear model that describes the frequency-domain relationship between the velocity of the cochlear partition $\left(V_{CP}\right)$ and the pressure difference $\left(P_{0}\right)$ across it. The cochlear partition comprises the organ of Corti and the overlying tectorial membrane, and $V_{CP}$ denotes the velocity of its center of mass. The pressure-velocity relation is characterized by a phenomenological admittance, Y, defined as $V_{CP}=YP_{0}$. (For simplicity, the implicit frequency dependence is not shown.) By applying mass conservation and Newton’s second law, we have that (see Appendix A and Ref. [17])

(10) $$\frac{1}{A}\frac{d}{dx}\left(A\frac{d\bar{P}}{dx}\right)+\alpha ZY\bar{P}=0.$$

In this equation, $\overset{‾}{P}$ is the pressure difference between the “upper” and “lower” fluid chambers [see Fig. 2(a)] averaged over their cross-sectional area (A). The term Z=iωM represents the “longitudinal” impedance due to the effective acoustic mass (M) of the fluids, and the complex function $\alpha =P_{0}/\overset{‾}{P}$ relates the driving pressure to the scalae-averaged pressure [18]; it depends on wavelength and on model geometry. For simplicity, we assume 1D wave propagation, which allows us to set α=1 and $\overset{‾}{P}=P_{0}$. The equations for 2D and 3D models are more complex and can be found in Appendix A. However, and as we will illustrate through numerical simulations [19], the qualitative implications derived from the 1D model remain applicable in more realistic 2D and 3D geometries.

For simplicity, our analytic treatment focuses on pressure, whose spatial amplification is similar to that of BM velocity [20]. The numerical simulations we show in Figs. 2(d) and 2(e) verify that the main results apply to BM velocity in a more complete model [21]. Importantly, the signal enhancement mechanism we elucidate here relies on active amplification that boosts the energy of sound-induced traveling waves more than that of internal noise; in these types of models, pressure amplification serves as a proxy of power amplification [22].

When we assume “reflectionless” boundary conditions at the apical and basal ends of the cochlea, the 1D Green’s function becomes (see Appendix)

(11) $$G\left(x,x^{\prime }\right)\approx \frac{1}{2i}\sqrt{\frac{A\left(x^{\prime }\right)}{A\left(x\right)}\frac{1}{k\left(x\right)k\left(x^{\prime }\right)}}\text{exp}\left[-i\int_{\text{min}\left(x,x^{\prime }\right)}^{\text{max}\left(x,x^{\prime }\right)}k\left(\hat{x}\right)d\hat{x}\right],$$

where k(x) is the complex wave number. The pressure response when the cochlea is driven from the stapes is simply [23]

(12) $$\bar{P}\left(x\right)=2i\bar{P}\left(0\right)k\left(0\right)G\left(x,0\right).$$

When the spatial gradients of cross-sectional area (A) and wave number (k) are gentle enough, the gain per unit length (g) is primarily determined by Im⁡(k), the imaginary part of k. Specifically, the log-gain per unit length can be approximated as dlog⁡(|G|)/dx~Im⁡(k). When Im⁡(k)>0, the gain per unit length is greater than 1, and the wave undergoes power amplification. On the other hand, when Im⁡(k)<0, the gain per unit length is less than 1, indicating attenuation. When the cochlear amplifier is inactive, Im⁡(k) is everywhere negative [Im⁡(k)<0]. But when the amplifier is maximally active, Im⁡(k) is positive basal to the characteristic place and negative apical to it. In other words, the wave peaks near the point $\overset{ˆ}{x}$ where Im⁡(k)=0, with Im⁡(k)>0 for $x<\overset{ˆ}{x}$ and Im⁡(k)<0 for $x>\overset{ˆ}{x}$ [24]. Importantly, waves cut off dramatically just apical to their characteristic place [see Fig. 2(b)], so that g≪1 for $x>\overset{ˆ}{x}$. In summary, whereas traveling waves are amplified (g>1) before they reach their characteristic place $\left(\overset{ˆ}{x}\right)$, they are rapidly attentuated (g≪1) as they pass beyond it. According to our analysis of the bidirectional amplifier [Eq. (9) and Fig. 1(c)], this arrangement fulfills the conditions necessary for boosting the SNR at the characteristic place.

### C. Amplification of narrow-band signals and noise

For the purposes of analyzing the effects of spatial amplification on SNR enhancement, we focus on a narrow frequency band centered around the signal frequency. Within an arbitrarily narrow frequency band, the internal noise can be approximated using spatially incoherent sinusoidal sources with randomly distributed amplitudes and phases. In particular, we assume that the noise sources are sinusoids with phases uniformly distributed on [0,2π) and magnitudes given by a non-negative random variable with mean μ and variance $\sigma^{2}$. Using this simplified noise model allows us to examine the impact of signal amplification on SNR without the confounding effects of bandwidth reduction induced by amplification. The rms noise pressure at a given location x can be approximated as

(13) $${\bar{P}}_{\text{noise }}\left(x\right)\approx \gamma \sqrt{\int_{0}^{L}{\left|G\left(x,x^{\prime }\right)\right|}^{2}dx^{\prime }},$$

where $\gamma^{2}=\mu^{2}+\sigma^{2}$. This expression represents the statistical average of the noise pressure implied by the amplitude distribution of incoherent sinusoidal sources. The integral $\int_{0}^{L}\;{\left|G\left(x,x^{\prime }\right)\right|}^{2}dx^{\prime }$ captures the propagation of noise power from basal and apical noise sources to the location x. Assuming that the wave number at the cochlear entrance [k(0)] is independent of cochlear amplification, we have that the SNR is [25]

(14) $$R\left(x\right)\propto \frac{\left|G\left(x,0\right)\right|}{\sqrt{\int_{0}^{x}{\left|G\left(x,x^{\prime }\right)\right|}^{2}dx^{\prime }+\int_{x}^{L}{\left|G\left(x,x^{\prime }\right)\right|}^{2}dx^{\prime }}},$$

where the two integrals, $\int_{0}^{x}\;{\left|G\left(x,x^{\prime }\right)\right|}^{2}dx^{\prime }\;$ and $\int_{x}^{L}\;{\left|G\left(x,x^{\prime }\right)\right|}^{2}dx^{\prime }$, represent the propagated contributions of noise sources located basal and apical to x, respectively. The values of these integrals, calculated using a previously developed 2D model (see figure caption and Appendix B for details), are shown in Fig. 2(c). The figure shows that at the characteristic place, the contribution of apical noise sources is negligible compared to that of basal noise sources.

Figure 2(d) depicts the differential effects of amplification on signal and internal noise in the 2D cochlear model for frequencies of 10 and 30 kHz. As expected from the analysis of the bidirectional amplifier, turning on the cochlear amplifier boosts the signal more than the internal noise near the characteristic place. This is evident in the plot, where the *in vivo* signal amplitude is larger than that of noise near the region of maximal BM response. (Note that signal and noise levels are normalized so that postmortem they are the same at the characteristic place.) However, as one moves basally away from the characteristic place towards the cochlear entrance, amplification becomes more pronounced for the internal noise compared to the signal. The differential effect of amplification on signal and internal noise highlights the selective enhancement of the signal relative to the noise at the characteristic place, where the cochlea achieves optimal sensitivity for sound detection.

### D. Amplification of broadband signals and noise

Figure 2(e) shows the enhancement factor as a function of distance along the cochlea when both signals and noise are broadband. In these simulations, signal and noise have white spectra over the frequency band spanning the full range of CFs represented by the cochlear model (4–70 kHz). Except near the cochlear entrance—where CF waves do not travel far enough to experience substantial amplification, to the point that there is no SNR enhancement even at CF [open symbols in Fig. 2(e)]—amplification substantially boosts the broadband SNR, by ~10 dB at the most sensitive locations. These results demonstrate that spatially restricted amplification produces a global increase in cochlear sensitivity to broadband sounds.

## IV. DISCUSSION

While the inner ear possesses astounding mechanical sensitivity, the origin of this sensitivity within the context of amplification has been largely overlooked. Indeed, the text-book view in the field is that the cochlear amplifier increases the sensitivity of hearing by boosting the mechanical vibrations that displace the stereocilia of the sensory neurons. This simplistic account ignores the fact that the sensitivity of a system depends on the internal noise [6,26]. The handful of previous attempts at relating cochlear amplification with (true) cochlear sensitivity (e.g., Refs. [27,28]) ignore the contributions of wave propagation, relying instead on nonequilibrium oscillator models whose relevance to cochlear mechanics remains uncertain.

We have shown here that established mechanisms of cochlear wave amplification produce significant signal enhancement. The mechanisms are analogous to human-made wave-based systems such as lasers and active transmission lines [11,29]. Indeed, the cochlear amplifier has been likened to the gain medium of a laser amplifier [30]. By amplifying different frequencies in different regions, the cochlea effectively employs narrow-band “laserlike” amplification to boost sensitivity to both narrow- and broad-band signals [Fig. 2(d)]. The waveguide structure of the cochlea allows it to act as an inhomogeneous transmission line in which the cutoff frequency changes with location [31]. In this way, waves within the operating frequency range are greatly attenuated before reaching the apical end (see also Refs. [32,33]). Consequently, the cochlea eliminates noise “build-up” due to scattering from the apical termination, an effect which can greatly degrade the performance of active transmission lines [29].

Our results also highlight the functional importance of the asymmetric shape of the cochlear filters (i.e., of the BM frequency response measured at each location). The cochlear filters have a steep high-frequency flank arising from the wave cutoff apical to the CF place. As a result, near-CF waves coming from more basal locations are amplified while those arising at more apical locations—where there are noise sources but no signal—are squelched. Thus, the steep wave cutoff underlies a peculiar form of spatial filtering of near-CF components, optimized to reject noise [34]. It is worth noting that the ear-horn-like geometry of the cochlea contributes significantly to this “optimized spatial filtering.” The tapered geometry facilitates the propagation of waves from the base to the apex, allowing for efficient signal propagation and amplification [23].

The strategy elucidated here for enhancing signal to noise within the cochlea is compelling because it is simple, robust, and consistent with established facts of active cochlear mechanics: first and foremost, that traveling waves are initially amplified and then dramatically attenuated as they propagate. But to what extent does this mechanism boost the sensitivity of hearing in actual practice? Although a precise answer to this question is currently out of reach—it requires details that are largely unknown and are likely to remain unknown for a long time (e.g., the power of the dominant intracochlear noise sources)—considerable insight can be gained by reviewing the empirical evidence in light of our findings. Specifically, Nuttall and colleagues [8] measured BM-velocity noise in the base of sensitive guinea-pig cochleae, carefully minimizing external interferance to ensure that the recordings were dominated by internal cochlear noise sources. At frequencies near CF, they found a BM mechanical noise floor approximately 15 dB below the BM vibration amplitude produced by tones at intensities corresponding to neural threshold. More recent recordings [35], in the apex of the mouse cochlea, yield similar results for the tectorial membrane [36]. In a nutshell, the experimental data suggest that the cochlear mechanical SNR, measured for narrow-band frequencies near-CF in response to threshold-level tones, is on the order of 15 dB. Strikingly, in our model amplification enhances the SNR of the BM responses by a similar amount [Fig. 2(e)]. In other words, our results suggest that without amplification cochlear mechanical responses to faint but detectable sounds would fall perilously close to the internal noise floor. Although there is no scarcity of factors that impact the neural encoding of sound—including hair-cell noise [37,38] and the stochastic nature of auditory-nerve firing [39]—our analysis suggests that spatially distributed cochlear amplification plays a central role in enhancing the sensitivity of hearing.

## Figures and Tables

**FIG. 3.**
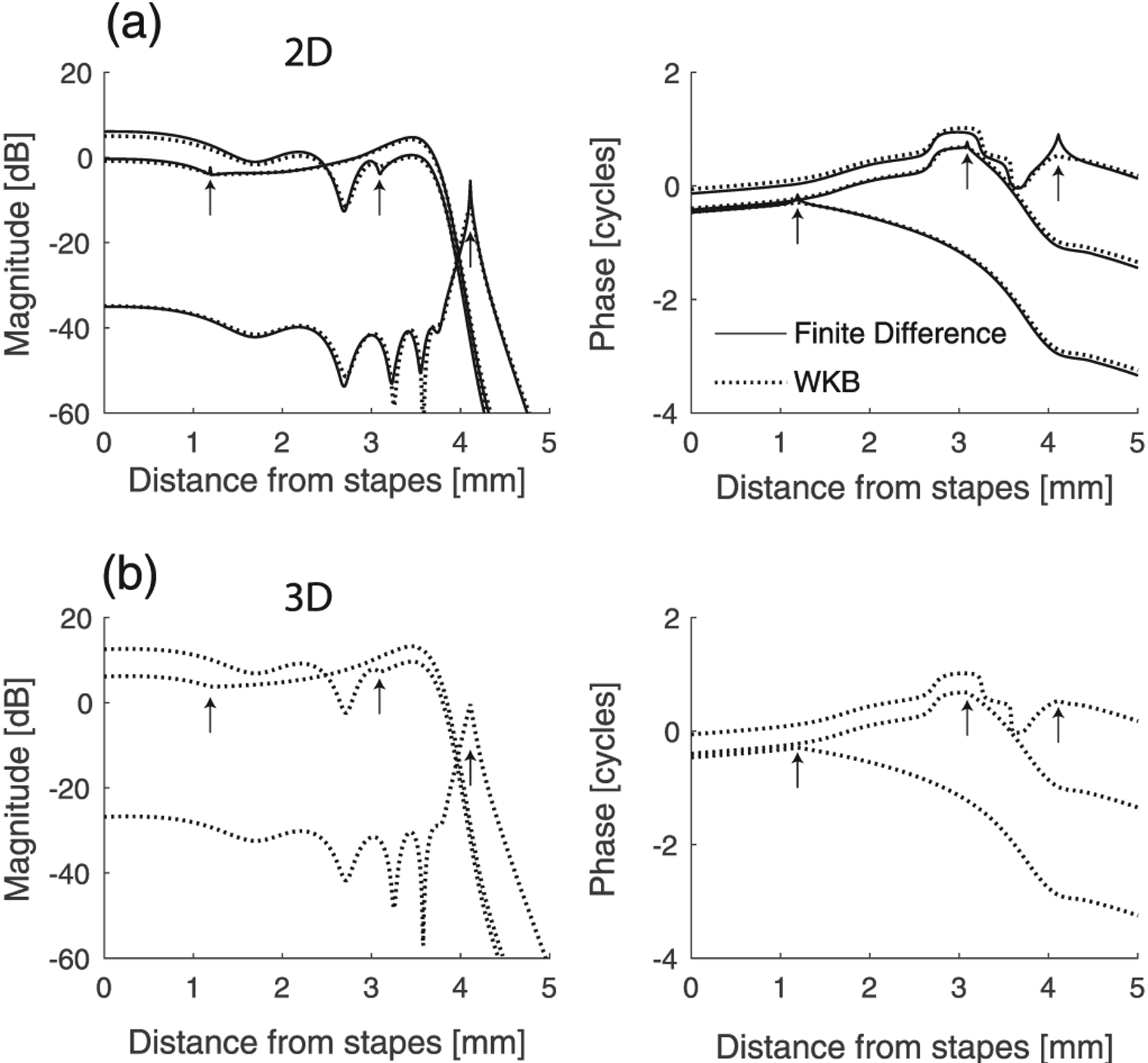
(a) Example of Green’s function for a 2D model with reflective basal boundary $\left(\left|R_{st}\right|\approx 0.14\right)$, calculated numerically in a finite-difference model (solid line) or with the WKB approximation [Eqs. (A11) and (A12), dashed lines]. The source locations for the various curves are indicated with vertical arrows; the source frequency is 10 kHz. (b) Approximate Green’s function for a simplified 3D model (see text).

## APPENDIX A: GREEN’S FUNCTIONS IN ONE, TWO, AND THREE DIMENSIONS

### 1. Equations of motion

The average pressure difference between the two scalae $\left(\overset{‾}{P}\right)$ and the velocity of the partition’s center of mass $\left(V_{CP}\right)$ are related by the well-known transmission-line equations:

(A1)
$$\frac{d\bar{P}}{dx}=-\frac{i\omega \rho }{A}U,\;\frac{dU}{dx}=-bV_{\text{CP}},$$

where U is the volume velocity of the fluids in the duct, A is the duct’s effective acoustic area, ρ is the fluid density, ω is the angular frequency, and b is the partition’s effective width. The partition velocity can be expressed as the product of the pressure difference across the tissue $P_{0}$ and a complex admittance $Y_{CP}$,

(A2)
$$V_{\text{CP}}=P_{0}Y_{\text{CP}}=\alpha \bar{P}Y_{\text{CP}},$$

where $\alpha =P_{0}/\overset{‾}{P}$ is the short-wave hydrodynamic factor. Combining Eqs. (A1) and (A2) we find the following expression for $\overset{‾}{P}$,

(A3)
$$\frac{1}{A}\frac{d}{dx}\left(A\frac{d\bar{P}}{dx}\right)+k_{x}^{2}\bar{P}=0,$$

where $k_{x}^{2}=\alpha ZY$ is the square of the complex wave number and Z=iωρ/A is the acoustic impedance of the scalae.

### 2. One-dimensional models

In 1D models, the pressure field is a function only of longitudinal distance from the stapes (x) so that $\overset{‾}{P}\equiv P_{0}$ and α=1. The Green’s function $G_{1D}\left(x,x^{\prime }\right)$ is the response to a unitary point pressure source at $x^{\prime }$, and hence can be expressed as

(A4)
$$\frac{1}{A}\frac{d}{dx}\left(A\frac{dG_{1\text{D}}}{dx}\right)+k_{x}^{2}G_{1\text{D}}=-\delta \left(x-x^{\prime }\right),$$

where δ is the Dirac delta function. Note that the pressure source has unit of pressure over length squared. We assume reflections boundary conditions and calculate $G_{1D}$ using the Wentzel-Kramers-Brillouin (WKB) approximation. To do so we make a change of variable in Eq. (A4). In particular, we introduce the acoustic distance χ,

(A5)
$$\chi =A\left(0\right)\int_{0}^{x}\frac{dx}{A\left(x\right)},$$

and define the corresponding wave number as $\overset{ˆ}{k}=Ak_{x}/A(0)$. Equation (A4) can be then rewritten as

(A6)
$$\frac{d^{2}G_{1\text{D}}}{d\chi^{2}}+{\hat{k}}^{2}G_{1\text{D}}=-\frac{A^{2}}{A^{2}\left(0\right)}\delta \left(x-x^{\prime }\right)\;=-\frac{A\left(x^{\prime }\right)}{A\left(0\right)}\delta \left(\chi -\chi^{\prime }\right).$$

In the χ domain, the 1D Green’s function is [31]

(A7)
$$G\left(\chi ,\chi^{\prime }\right)=\frac{1}{2i}\sqrt{\frac{1}{\hat{k}\left(\chi \right)\hat{k}\left(\chi^{\prime }\right)}}\text{exp}\left[-i\int_{\text{min}\left(\chi ,\chi^{\prime }\right)}^{\text{max}\left(\chi ,\chi^{\prime }\right)}\hat{k}\left(\hat{\chi }\right)d\hat{\chi }\right]\text{.}$$

Accounting for the source amplitude in the χ domain [Eq. (A6)], and converting the solution back into the x domain, yields

(A8)
$$G_{1\text{D}}\left(x,x^{\prime }\right)=\frac{1}{2i}\sqrt{\frac{A\left(x^{\prime }\right)}{A\left(x\right)}\frac{1}{k\left(x\right)k\left(x^{\prime }\right)}}\text{exp}\left[-i{\int^{}}_{\text{min}\left(x,x^{\prime }\right)}^{\text{max}\left(x,x^{\prime }\right)}k\left(\hat{x}\right)d\hat{x}\right].$$

### 3. Two-dimensional and simplified three-dimensional models

A tapered 2D “box” model can be interpreted physically as a model where the cross-sectional area of the duct is a rectangle with constant width and varying height, while the partition spans the entire cochlear width and moves up and down as a piston (“wall-to-wall carpeting,” see Ref. [31]). The equations for a 2D model are approximately valid for a 3D model where the cochlear duct and partition have circular cross-sectional shapes. When the radius of the partition is sufficiently small, the pressure can be approximated as a function of distance from the stapes (x) and radial distance from the partition center (r). With these approximations the 3D model is effectively 2D in cylindrical coordinates [40,41].

Importantly, although the equations for a 2D box model are valid for a 3D cylindrical model, the parameters and their spatial gradients in the two models are different [see also 22]. While the partition admittance can be strategically chosen so that the wave number $k_{x}$ is the same in 2D and 3D [42], the spatial gradient of the cross-sectional area A (which determines the important geometric pressure gain factor [23]) differs in the two models. In the tapered box model A∝H, while in the 3D cylindrical model $A\propto H^{2}$, where H is the scala height (or radius).

Keeping in mind these important caveats, we now proceed to heuristically determine the reduced 2D Green’s function ${\overset{‾}{G}}_{2D}\left(x,x^{\prime }\right)$ which describes the scalae-average pressure at x resulting from a 2D source placed at the center of the partition (i.e., at y=0). Following [31], we note that the 2D reduced Green’s function must obey the following relation:

(A9)
$$\frac{1}{A}\frac{d}{dx}\left[A\frac{d{\bar{G}}_{2\text{D}}\left(x,x^{\prime }\right)}{dx}\right]+k_{x}^{2}{\bar{G}}_{2\text{D}}\left(x,x^{\prime }\right)=ℱ\delta \left(x-x^{\prime }\right),$$

where ℱ is a function to be determined that accounts for the fact that the source, unlike in the 1D model, is also two dimensional. Following the results of Ref. [31] obtained in a box model of constant cross-sectional area, we have that ${\left.ℱ\right|}_{x^{\prime }}\propto \alpha \left(x^{\prime }\right)$. Because in our tapered model the area changes with location, we further need to figure out if there are systematic differences between 1D, 2D, and 3D sources that change with the cross-sectional area. In this regard, we note that a 2D point source is $s_{2D}=\delta \left(x-x^{\prime }\right)\delta (y)$ while a one-dimensional source is $s_{1D}=\delta \left(x-x^{\prime }\right)$. Their respective source strengths, averaged over the cross sectional area of a two-dimensional model, are a factor of $H\left(x^{\prime }\right)$ larger in 1D than in 2D [43]. Likewise a 3D source is $s_{3D}=\delta \left(x-x^{\prime }\right)\delta (y)\delta (z)$, whose strength is $A\left(x^{\prime }\right)$ smaller than a 1D one.

Based on these consideration, and further noting that $A\left(x^{\prime }\right)\propto H\left(x^{\prime }\right)$ in 2D (so that we can write equations that are valid in 2D and 3D models), we conclude that $ℱ\approx \alpha \left(x^{\prime }\right)/A\left(x^{\prime }\right)$:

(A10)
$${\bar{G}}_{2\text{D}}\left(x,x^{\prime }\right)\approx \frac{\alpha \left(x^{\prime }\right)}{A\left(x^{\prime }\right)}G_{1\text{D}}\left(x,x^{\prime }\right).$$

We now define ${\overset{ˆ}{G}}_{2D}\left(x,x^{\prime }\right)=G_{2D}\left(x,0,x^{\prime },0\right)$, where $G_{2D}\left(x,y,x^{\prime },y^{\prime }\right)$ is the “true” 2D Green’s function, i.e., the function that describes the pressure response at x,y to a unit point source at $x^{\prime },\;y^{\prime }$. Exploiting the definition of α, we have that

(A11)
$${\hat{G}}_{2\text{D}}\left(x,x^{\prime }\right)\approx \frac{\alpha \left(x\right)\alpha \left(x^{\prime }\right)}{2i}\sqrt{\frac{1}{k\left(x\right)k\left(x^{\prime }\right)A\left(x^{\prime }\right)A\left(x\right)}}\times \text{exp}\left[-i\int_{\text{min}\left(x,x^{\prime }\right)}^{\text{max}\left(x,x^{\prime }\right)}k\left(\hat{x}\right)d\hat{x}\right],$$

where it can be appreciated that ${\overset{ˆ}{G}}_{2D}\left(x,x^{\prime }\right)={\overset{ˆ}{G}}_{2D}\left(x^{\prime },x\right)$. The same Green’s function holds for the simple 3D model $\left[G_{3D}\left(x,x^{\prime }\right)\approx {\overset{ˆ}{G}}_{2D}\left(x,x^{\prime }\right)\right]$, keeping in mind the caveats regarding the cross-sectional areas in the two models.

### 4. Nonideal boundary conditions and numerical solutions

The solutions for the Green’s functions shown above were obtained under the assumption of no significant scattering from the basal and apical boundaries. While this is a good approximation for the apical boundary—because traveling waves are dramatically attenuated before reaching it [32]—the same is not true for the basal boundary at the stapes, where any impedance mismatch at the boundary with the middle ear has the effect of backscattering a significant fraction of wave power [44]. When the wave frequency is sufficiently smaller than the CF near the stapes we can assume long-wave behavior near the stapes. In this case, we can easily include the effect of wave reflection and calculate the WKB approximation for this nonidealized Green’s function

(A12)
$${\tilde{G}}_{2\text{D}}\left(x,x^{\prime }\right)={\hat{G}}_{2\text{D}}\left(x,x^{\prime }\right)+R_{\text{st}}{\bar{G}}_{2\text{D}}\left(0,x^{\prime }\right)\alpha \left(x\right)\sqrt{\frac{A\left(0\right)k\left(0\right)}{A\left(x\right)k\left(x\right)}}\times \text{exp}\left[-i\int_{0}^{x}k\left(\hat{x}\right)d\hat{x}\right],$$

where $R_{st}$ is the complex reflectance of the stapes [44]. The second term on the right side of Eq. (A12) represents a wave traveling from the base to the apex, generated by the pressure reflected from the stapes $\left[R_{st}{\overset{‾}{G}}_{2D}\left(0,x^{\prime }\right)\right]$.

### 5. Numerical and semianalytical calculations

We cross-checked the quality of our calculations by comparing the 2D WKB approximation of the Green’s function against numerical calculations performed in a tapered 2D finite-difference model [45,46], some of which are shown in Fig. 3(a). Because calculating the WKB approximation for α requires iterative methods that introduce various inaccuracies, we calculated α numerically, driving the finite-difference model from the stapes. Figure 3(b) shows the WKB solution for the Green’s function of a 3D model with the same wave number (k) and height (H) as the 2D model in Fig. 3(a). While the agreement between the WKB approximation and the numerical solution is generally excellent, the WKB approximation can introduce significant errors (due to the nonuniqueness of the WKB solution in the cutoff region [47]), rendering the calculations noisy, especially at high frequencies. For this reason, in the main text we present results obtained using the 2D finite-difference model—the differences between 2D and 3D models are relatively minor, although it is worth mentioning that in 3D the enhancement factors are slightly larger thanks to the more dramatic tapering of the cross-sectional area in 3D than in 2D models.

## APPENDIX B: MODELING DETAILS

We performed all calculations using an “overturned model” of the mouse cochlea [41], whose parameters are the same as those used in Ref. [48]. In this model, unlike in classic models where the organ of Corti does not deform, the transverse (up-down) velocity of the center of mass is $V_{CP}=\left(V_{BM}+V_{top}\right)/2$, where $V_{BM}$ and $V_{\text{top}}$ indicate the velocity of the bottom (BM) and the top-side (the reticular lamina and tectorial membrane) of the organ of Corti—their differential velocity is $V_{\text{int}}=V_{\text{top}}-V_{BM}$. Postmortem, $V_{BM}$ and $V_{\text{top}}$ are similar, so that to a first approximation $V_{\text{int}}\approx 0$ in a passive cochlea, while $V_{\text{int}}\neq 0$ *in vivo*. The center-of-mass velocity can be rewritten in the compact form $V_{CP}=V_{BM}+V_{\text{int}}/2$, where $V_{\text{int}}$ is attributed to the piezoelectric action of the OHCs and is effectively the (velocity) source of wave amplification in the model.

Because the BM stiffness is about one order of magnitude larger than that of the structures surrounding the OHCs, OHC forces produce large displacements of the top side of the organ of Corti while having secondary effects on local BM motion [49,50]. We therefore assume that internal OHC forces have negligible effects on BM motion so that the mechanical admittance of the $BM\left(Y_{BM}=V_{BM}/P_{0}\right)$ is constant, independent of whether the cochlear amplifier is turned “on” or “off.” For simplicity, we also assume that $Y_{BM}$ represents the admittance of a damped harmonic oscillator. By exploiting the relationships between $V_{CP},\;V_{BM}$, and $V_{\text{int}}$, we can express the admittance of the organ of Corti admittance as $Y_{CP}=Y_{BM}\left(1+\frac{1}{2}V_{\text{int}}/V_{BM}\right)$. Following previous results, we assume that *in vivo* at low sound levels $\frac{1}{2}V_{\text{int}}/V_{BM}\approx i\beta \tau$, where β=f/CF is normalized frequency and τ is a (real) constant. Following Ref. [23], we assume that $Y_{CP}$ is scaling symmetric (i.e., a function only of normalized frequency, β) throughout the cochlea.
